# ROR1, an embryonic protein with an emerging role in cancer biology

**DOI:** 10.1007/s13238-014-0059-7

**Published:** 2014-04-22

**Authors:** Nicholas Borcherding, David Kusner, Guang-Hui Liu, Weizhou Zhang

**Affiliations:** 1 Department of Pathology, College of Medicine, University of Iowa, Iowa City, IA 52242 USA; 2 Department of Molecular and Cellular Biology, College of Medicine, University of Iowa, Iowa City, IA 52242 USA; 3 National Laboratory of Biomacromolecules, Institute of Biophysics, Chinese Academy of Sciences, Beijing, 100101 China; 4 Beijing Institute for Brain Disorders Brain Tumor Center, Beijing, 100101 China

**Keywords:** ROR1, embryogenesis, cancer, immunotherapy

## Abstract

Receptor tyrosine kinase-like orphan receptor 1 (ROR1) is a member of the ROR family consisting of ROR1 and ROR2. RORs contain two distinct extracellular cysteine-rich domains and one transmembrane domain. Within the intracellular portion, ROR1 possesses a tyrosine kinase domain, two serine/threonine-rich domains and a proline-rich domain. RORs have been studied in the context of embryonic patterning and neurogenesis through a variety of homologs. These physiologic functions are dichotomous based on the requirement of the kinase domain. A growing literature has established ROR1 as a marker for cancer, such as in CLL and other blood malignancies. In addition, ROR1 is critically involved in progression of a number of blood and solid malignancies. ROR1 has been shown to inhibit apoptosis, potentiate EGFR signaling, and induce epithelial-mesenchymal transition (EMT). Importantly, ROR1 is only detectable in embryonic tissue and generally absent in adult tissue, making the protein an ideal drug target for cancer therapy.

## Introduction

**Figure 1:**
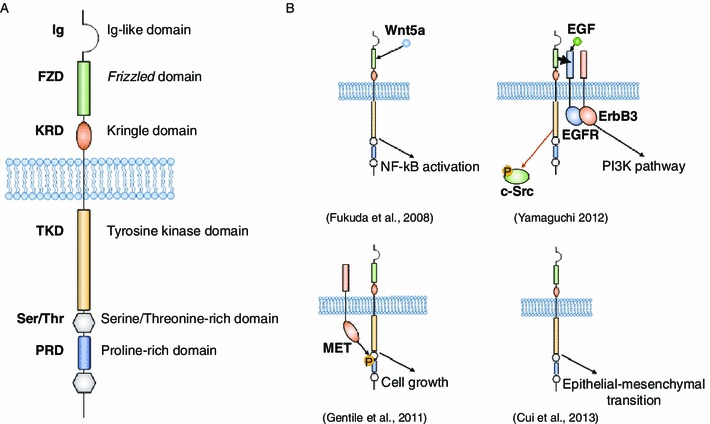
**ROR1 structure and signaling in cancer**. (A) Human ROR1 consists of an immunoglobulin-like domain (IG), two cysteine-rich domain, *frizzled* (FZD) and kringle domain (KRD). On the intracellular side, ROR1 possesses a tyrosine kinase domain (TKD), two serine/threonine-rich domains (Ser/Thr), and a proline-rich domain (PRD). (B) ROR1-mediated signaling has been reported in a number of cell lines. Wnt5a, the ligand of ROR1, increased NF-kB activation in HEK293 cells expressing ROR1. In lung adenocarcinoma cell lines, ROR1 is able to phosphorylate c-SRC and through allosteric interaction of the FZD with EGFR magnify the EGF-induced signaling. Alternatively, in lung carconoma and gastric carcinoma cell lines, ROR1 is phosphorylated by MET; the silencing of ROR1 impairs cell growth. In MDA-MB-231 breast cancer cells, ROR1 expression is highly associated with EMT genes and the silencing of ROR1 reduces the ability of MDA-MB-231 cells to form metastic foci

ROR1 and ROR2 are transmembrane proteins within the receptor tyrosine kinase (RTK) family. ROR1/2 were initially discovered in a neuroblastoma cell line in a PCR screen for receptor tyrosine kinases and were formerly named as neurotrophic tyrosine kinase receptor-related (NTRKR1/2). Human ROR1/2 have 58% amino acid identity and are closely related to MUSK and Trk family receptors (Masiakowski and Carroll, 1992; Forrester et al., 1999). Both genes encode proteins with a predicted molecular weight of 104 kDa, but ROR1 has multiple N-glycosylation sites that generate post-translationally modified ROR1 at 130 kDa. These N-glycosylation sites are necessary for the trafficking of ROR1 to the membrane and in turn the function of ROR1 (Kaucká et al., 2011). The structure of human ROR1/2 consists of an extracellular immunoglobulin-like (Ig) domain at the amino-terminus, followed by a cysteine-rich domain known as a Frizzled domain (FZD), and then a Kringle domain (KRD) into a transmembrane domain (Fig. 1A). The FZD domain is seen in the Smoothened and Frizzled-family receptors, as well as carboxypeptidase Z, collagen α1 XVIII, and low-density lipoprotein receptor-related proteins (LRP) and consists of 10 conserved cysteine residues and five corresponding disulfide bonds. The FZD is thought to mediate receptor-ligand interaction (Roszmusz et al., 2001; Forrester et al., 2004; Mikels and Nusse, 2006). Both ROR1 and to a greater extent in the literature, ROR2, have been shown to bind Wnt5a, a non-canonical Wnt via the FZD (Oishi et al., 2003; Mikels and Nusse, 2006; Fukuda et al., 2008; Paganoni et al., 2010). The KRD is a highly-folded, cysteine-rich domain that mediates in protein-protein and protein-ligand interaction in coagulation proteins, apolipoproteins, and hepatocyte growth factor (Stephens et al., 1992; Mizuno et al., 1994; Mathews et al., 1996). The cytoplasmic portion of human ROR1/2 has a tyrosine kinase domain (TKD), followed by a Serine/Threonine-rich domain (Ser/Thr), a proline-rich domain (PRD), and a second Ser/Thr domain at the carboxy-terminus (Fig. 1A). The functionality of the tyrosine kinase domain (TKD) of RORs has been debated in the literature. Early studies showed strong autocatalytic kinase activity for ROR2, while ROR1 possessed weak to moderate kinase activity (Masiakowski and Carroll, 1992; Oishi et al., 1999). More recently, ROR1 TKD was shown to be sufficient to phosphorylate c-SRC in NIH3T3 cells (Yamaguchi et al., 2012). Conversely, another study concluded that ROR1 is a pseudokinase as ROR1 did not show any kinase activity in COS-7 cells (Gentile et al., 2011). The ROR families are one of the most divergent within the receptor tyrosine kinase family, containing only 21 of the 40 consensus residues in other TKDs described by Hanks and Quinn (Hanks et al., 1988). Notably, ROR1 possesses substitutions at C482G, K614R and L634F, that should modulate ATP binding and kinase function (Hanks et al., 1988; Masiakowski and Carroll, 1992). The ROR family of proteins are evolutionally conserved and share a high level of homology between orthologs in *Mus musculu*s, *Caenorhabditis elgans*, *Xenopus laevevis*, *Drosophila melnogaster*, *Apylasia californica*, and *Gallus gallus* (Wilson et al., 1993; Forrester et al., 1999; Oishi et al., 1999; McKay et al., 2001; Hikasa et al., 2002; Stricker et al., 2006). CAM-1 is the singular ROR1/2 ortholog in *C. elegans* and shares a greater amino acid identity to ROR1, but lacks the PRD and the second Ser/Thr domain. DROR, a structural intermediate of the ROR and TRK receptor family, and DRNK are the *Drophosilia* orthologs and lack the extracellular Ig domain and the intracellular PRD and Ser/Thr domains (Wilson et al., 1993; Oishi et al., 1997). The conservation of RORs across species underlies the importance of the ROR family through a number of processes during evolution.

## ROR1/2 functions within development

A series of studies that utilized *in situ* hybridization and mutant knockout characterizations in mice have implicated RORs in the context of skeletal, cardiorespiratory, and neurological development. The expression patterns of mROR1 and mROR2 in embryos partially overlap, namely in facial development, pharyngeal arches, nasal processes, and much of the other derivatives of neural crest cells. In general mROR1 is restricted to the cephalic mesenchyme and neural crest cells, while mROR2 is expressed more broadly in both neural and non-neural cells throughout development. Within the limb, a low level of mROR1 is detected at the proximal portion of the limb bud, while mROR2 expression extends throughout the mesenchyme of the limb. Later in development, strong expression of mROR2 is seen within the perichondrium of the developing digits, while mROR1 expression is seen in the necrotic and interdigital zones (Al-Shawi et al., 2001; Matsuda et al., 2001). The expression of mROR2 within the subset of chondrocytes at the growth plate and perichondrium suggests a functional role within the development of bones with cartilaginous anlage (DeChiara et al., 2000). The potential role of mROR2 in limb/skeletal formation is underscored by the identification of mutations in hROR2. Mutations of hROR2 in the intracellular Ser/Thr domains, PRD or nonsense mutations have been linked to the dominant Brachydactyly Type B, characterized hypoplasia and/or aplasia of the hands and feet (Oldridge et al., 2000). hROR2 mutations in the CRD, KRD, TKD, and residues immediately following TKD have also been associated with Robinow syndrome, a recessive short-limbed dwarfism (Afzal et al., 2000; van Bokhoven et al., 2000). In late stages of mouse development, the expression of mROR1 and mROR2 is seen within the heart and alveoli of the lungs. Mice with homozygous knockout of *mROR2* exhibit shortened limbs, cyanosis, septal defects of the heart and die within six hours of birth due to respiratory defect (Takeuchi et al., 2000). Likewise, *mROR1*^−/−^ mice have perinatally lethal defects due to respiratory dysfunction; however, these mice do not exhibit the pronounced heart or skeletal abnormalities. When researchers generated a *mROR1*/*mROR2*-deficient mouse, they found a more severe phenotype than *mROR2*^−/−^ alone, leading the authors to conclude that mROR1/2 have a redundant, yet none overlapping function in development (Nomi et al., 2001). In late stages of embryonic development, the expression of mROR2 is sustained in the hippocampus and caudate putamen, but mROR1 is no longer detectable (Matsuda et al., 2001). The temporal and localized expression of both mRORs within the developing nervous system underscores their function in neurogenesis.

CAM-1, the only ROR homolog in *C. elegans*, has been extensively studied in neuronal cell migration, asymmetric division, and axonal outgrowth. Mutations within the cysteine-rich FZD and truncating mutations before the kinase domain result in the inhibition of axonal outgrowth, suggesting a ligand-mediated function, but kinase activity may be dispensable. However, kinase activity is necessary for asymmetric neuronal division (Forrester et al., 1999). Additionally mutations in CAM-1 lead to defects specifically in canal-associated neurons. CAM-1 is considered to be a negative regulator of canonical Wnt signaling; excess EGL-20, a canonical Wnt ligand, mirrors the neuronal phenotype of CAM-1 mutants. The extracellular FZD is necessary and sufficient to mediate the antagonistic role for canonical Wnt during neuronal migration (Forrester et al., 2004; Green et al., 2007). The role of RORs in neuronal migration is seen in others species; the *Xenopus* homolog xROR2 inhibits convergent extension of the neuroectoderm via non-canonical Wnt signaling (Hikasa et al., 2002). RORs have also been indicated in synapse formation. The *Aplysia* ROR homolog clusters on bag neuron cells suggesting organization of functional sites or synapses in *Aplysia californica* (McKay et al., 2001). Down regulation of ROR1 or ROR2 via small interfering (si) RNA decreases synaptogenesis in primary mouse embryonic neuronal cultures. mROR1 and mROR2 can form heterodimers within human embryonic kidney (HEK) 293 cells that bind to the putative ligand Wnt5a. Treating the primary embryonic cells with Wnt5a increases synapse number in a dose dependent manner, suggesting a functional role of Wnt5a-ROR1/2 in synapse formation (Paganoni et al., 2010).

## ROR1 in cancer

While ROR1 expression is present during normal embryonic and fetal development, it is absent within most mature tissues. A low level of ROR1 expression is seen in adipose tissue and to a lesser degree in the pancreas, lung, and a subset of intermediate B cells (Baskar et al., 2008; Hudecek et al., 2010; Bicocca et al., 2012). However, the expression of ROR1 has been seen in numerous blood and solid malignancies. This differential expression pattern, low ROR1 expression in adult tissue and high expression in cancer have led investigators to examine the functional advantage conferred to cancer by ROR1 expression and to explore the use of immune-based therapies against ROR1 for targeting cancer cells. (Baskar et al., 2008; Barna et al., 2011; Zhang et al., 2012a; Zhang et al., 2012b; Daneshmanesh et al., 2013)

### ROR1 in blood malignancies

Strong expression of ROR1 was initially identified in B-Cell chronic lymphocytic leukemia (CLL). Primary CLL cells express high levels of ROR1, but not ROR2, and this expression was not modulated by the incubation with CD40 or IL-4 (Baskar et al., 2008). A second research group independently identified ROR1 in CLL after *ex vivo* transduction of CD40 ligand (CD154) and autologous infusions of the transduced cells into advanced stage CLL patients. This transduction reversed the characteristic immunosuppression of CLL and resulted in the generation of antibodies against ROR1. Furthermore, anti-ROR1 immunoglobulins were shown to bind specifically with CLL cells, while being unreactive towards peripheral blood mononuclear cells (PBMCs) from CLL patients and healthy donors (Fukuda et al., 2008). The expression of ROR1 increases through the progression of CLL. ROR1 is not only a biomarker for CLL, but it may serve as a potential prognostic indicator (Daneshmanesh et al., 2013). The constitutive phosphorylation of STAT3, a hallmark of CLL, has been shown to bind multiple sites in the ROR1 promoter. In addition, the expression of ROR1 could be induced by IL-6 in a STAT3-dependent and dose-dependent manner (Frank et al., 1997; Li et al., 2010). As Wnt5a was shown to bind ROR1 in HEK293 cells, resulting in NF-κB activation in a reporter construct, ROR1 may be responsible for controlling self-expression (Fig. 1B) (Fukuda et al., 2008). Since the discovery of the elevated expression of ROR1 in CLL, increased levels of ROR1 have been described in a variety of hematological malignancies, including acute lymphocytic leukemia (ALL), non-Hodgkin lymphomas (NHL), and myeloid malignancies (Baskar et al., 2008; Daneshmanesh et al., 2008; Barna et al., 2011; Daneshmanesh et al., 2013). Specifically for NHLs, when compared to PBMCs, ROR1 mRNA and/or protein are elevated in all or a subset of primary samples of mantle cell lymphoma (MCL), marginal zone lymphoma (MZL), diffuse large B-cell lymphoma (DLBCL), and follicular lymphoma (Barna et al., 2011; Daneshmanesh et al., 2013). A high level of ROR1 expression is seen in ALL patients, specifically those with the t(1;19)(q23;p13) translocation. ROR1 is identified to be important for the survival of ALL cells with t(1;19)(q23;p13) translocation when using an siRNA library for the tyrosine kinome to screen critical tyrosine kinases for ALL pathogenesis (Bicocca et al., 2012). Examination of the expression levels in normal and B cells at early developmental stages lack ROR1 expression; however, normal B cells in an intermediate stage of development (large/small pre-BII and immature B cells) show relatively high levels of ROR1 expression, which is absent within mature B cells (Hudecek et al., 2010; Bicocca et al., 2012). Interestingly, t(1;19) ALL cells are generally characterized as residing in late stages of B cell differentiation, such as large/small pre-BII. Therefore, the presence of ROR1 in t(1;19) ALL cells may represent an arrested intermediate stage during B cell maturation. Furthermore, these authors proposed a model for normal B cell development, wherein ROR1 expression is upregulated at the pre-BII large stage. This allows for the maintenance of pro-survival signaling through MEK/ERK activation, which would be otherwise absent during pre-BCR internalization (Bicocca et al., 2012).

### ROR1 in solid malignancies

High expression of ROR1 is also observed in a wide variety of solid malignancies. Tissue microarray analysis showed strong staining of ROR1 in 30% or greater of primary samples in colon, lung, and pancreatic cancers. However, moderate staining is detected in the majority of ovarian, lymphoma, skin, testicular, uterine, prostate, and adrenal cancers (Zhang et al., 2012b). An RNAi-based screening in HeLa cells identified an important role of ROR1 in regulating apoptosis (MacKeigan et al., 2005). In lung adenocarcinoma, NKX2-1 (TITF1) has been shown to drive ROR1 expression and the subsequent increase in ROR1 has two distinct proposed functions, including the potentiation of EGF ligand-induced EGFR signaling and the phosphorylation and activation of c-Src regardless of ligand induction (Fig. 1B) (Yamaguchi et al., 2012). As previously mentioned, researchers have seen mild to moderate autophosphorylation of ROR1 (Masiakowski and Carroll, 1992; Oishi et al., 1999). However, the autophosphorylation is not detected using immunoprecipitated ectopic ROR1 in COS-7 cells, leading the authors to conclude ROR1 is a pseudokinase. In the same study, phosphorylated ROR1 was identified in a number of cell lines. This phosphorylation was found to be mediated by MET (HGFR), but not EGFR or ERBB2 (Fig. 1B). Within non-small cell lung cancer cells (NCI-H1993), silencing of ROR1 disrupts the ability to escape anoikis, anchorage-dependent programmed cell death, and decreased primary tumor growth when the cells are transplanted into nude mice (Gentile et al., 2011). In another study, Wnt5a, but not Wnt3a, binds to ROR1 that in turn recruits Frizzled 4 (FZD4) through FZD4’s CRD. The transient localization allows FZD4-associated glycogen synthase kinase 3β (GSK3β) to phosphorylate ROR1 on Ser/Thr residues (Grumolato et al., 2010). Therefore, ROR1 may serve as a common node for kinase phosphorylation and allow for subsequent pathway activation through adaptor/effector protein recruitment.

Segueing into breast cancer, ROR1 has been shown to be expressed in human neoplastic breast cancer cells, while remaining absent within stromal cells (Zhang et al., 2012a). Furthermore, high expression of ROR1 is associated with higher grade and more aggressive disease. ROR1, when stimulated by recombinant Wnt5a, interacts with casein kinase1ε (CK1ε), whose subsequent interaction with phosphoinositide 3-kinase (PI3K) results in the phosphorylation of AKT and CREB (Zhang et al., 2012a). High levels of ROR1 expression in patients and cell lines are associated with genes involved in epithelial-mesenchymal transition (EMT) such as ZEB1 and vimentin, and inversely associate with adherent junction proteins (Fig. 1B). Silencing of ROR1 in triple-negative-derived cell lines reduced EMT genes, SNAI1, SNAI2, ZEB1, and vimentin. In MDA-MB-231 cells, a triple negative breast cancer cell line, knockdown of ROR1 by small hairpin (sh) RNA reduces *in vitro* cell migration and bone and lung foci size in xenografts (Cui et al., 2013).

Although protein levels of ROR1 are low or not detectable within the kidney of healthy individuals, *ROR1* mRNA can be detected in 81.3% of tissue samples and 94% of PBMCs samples from renal cancer patients as determined by RT-PCR (Rabbani et al., 2010). Furthermore, PBMCs from renal cancer patients showed significantly higher ROR1 expression, compared to healthy controls. While these findings suggest that ROR1 expression is a hallmark of renal cancer, it is important to note that the protein levels of ROR1 are not measured in this study. The expression of ROR1 is detected in multiple melanoma cell lines, as assessed by RT-PCR, Western blot, and flow cytometry. Silencing ROR1 in all melanoma cell lines tested induces apoptosis (Hojjat-Farsangi et al., 2013). An interesting paradigm has been suggested in melanoma cell lines. Transcriptomic analysis of melanoma cell lines reveals that the expression of ROR1 is associated with a proliferative signature, but also correlates with a non-invasive phenotype. Treatment of two poorly invasive cell lines with recombinant Wnt5a leads to a significant decrease in ROR1 expression and overall protein level. Interestingly, silencing ROR1 leads to an increase in Wnt5a and ROR2 expression, supporting a more invasive phenotype of melanoma cells. The ROR1 and ROR2 expression seems to be differentially regulated also under hypoxic conditions that leads to decreased ROR1 expression and increased expression of ROR2 (O’Connell et al., 2013).

### ROR1, a target of immunotherapies

The discovery of ROR1 expression in CLL and other cancers has informed a diverse array of research on immuno-based strategies to target ROR1 (Baskar et al., 2008; Fukuda et al., 2008). In CLL, there are an estimated 1–7 × 10^4^ ROR1 molecules on the surface, which is 10–100 folds lower than the conventional targets of monoclonal antibody (mAb) therapies. Thus, the results from *ex vivo* analyses of mAbs against ROR1 in CLL have been mixed. A number of studies have reported anti-ROR1 induces ROR1 internalization (Baskar et al., 2008; Yang et al., 2011; Baskar et al., 2012; Daneshmanesh et al., 2013). Low levels of antibody dependent cellular cytotoxicity (ADCC) and even lower complement-dependent cytotoxicity (CDC) have been reported in primary CLL samples and MCL cell lines treated with anti-ROR1 mAbs (Yang et al., 2011; Baskar et al., 2012). In contrast, researchers developed several mAbs directed against the FZD and KRD of the extracellular region that have demonstrated high levels of cytotoxicity in primary CLL samples. These mAbs exhibit a significantly greater cytotoxicity than primary CLL samples treated with rituximab, an FDA-approved mAb against CD20 (Daneshmanesh et al., 2013). Inhibiting ROR1 by a mAb reduces metastatic foci in lungs assessed by bioluminescence and histology with xenografts of MDA-MB-231 breast cancer cells (Cui et al., 2013). Within melanoma cell lines, treatment with anti-ROR1 mAb results in varying degrees of apoptosis, between 4% and 54%, which is dependent upon the specific anti-ROR1 mAb and melanoma cell lines. This can be attributed partially to the antibody-mediated complement-dependent cytotoxicity (CDC) or ADCC. Importantly treatment of anti-ROR1 mAb, either directly or through CDC or ADCC, has no effect on apoptosis in the ROR1-negative cell line T47D (Hojjat-Farsangi et al., 2013). Research into immunotoxin therapies has been moving forward as well. The Fv region of an anti-ROR1 monoclonal antibody has been fused to PE38, a modified exotoxin from *Pseudomonas*. The immunotoxin exhibits similar specificity for ROR1 in MCL cell lines, but has a higher rate of dissociation from the receptor after internalization, which may be a limiting factor for translation into clinical studies (Baskar et al., 2012). Another approach also has been developed by the transduction of T cells with a ROR1-chimeric antigen receptor (ROR1-CAR) from healthy individuals to CLL patients. These ROR1-CAR T cells can recognize tumors cells and lyse primary CLL and MCL cells. The limitation to this approach is observed in the same study. While there is no ROR1 expression in hematopoietic progenitors, ROR1 is expressed in the median stages of B-cell maturation, in thymus-derived CD8^+^ and CD4^+^ T cells, adipose tissue and adult lungs. This warrants further study of its toxic effect for clinical usage (Hudecek et al., 2010; Bicocca et al., 2012).

## Prospective

ROR1 is expressed in a number of malignancies with low levels of expression in normal adult tissue. Much like the physiological functions of ROR1, ROR1 in cancer can have kinase activity-dependent or -independent function, which could be a result of tissue specific expression of co-receptor or effector proteins. The induction of apoptosis with ROR1 knockdown, EGFR signaling potentiation and ROR1-mediated upregulation of EMT genes support the notion that ROR1 plays an important role in cancer progression rather than just a bystander. Further research is required to elucidate the tumor-specific mechanisms of ROR1 overexpression and the contribution of ROR1 to initiation and progression of cancer. Furthermore, as an oncofetal protein with absence of expression in most adult tissue, ROR1 represents an ideal druggable target for cancer therapy.

## Abbreviations

ADCC: antibody dependent cellular cytotoxicity
CDC: complement dependent cytotoxicity
FZD: frizzled domain
KRD: kringle domain
mAb: monoclonal antibody
MUSK: muscle specific kinase
PRD: proline-rich domain
ROR1/2: receptor tyrosing kinase-like orphan receptor 1/2
TKD: tyrosine kinase domain
